# Factors Associated With Feeling Ashamed of Disclosure of HIV‐Positive Status Among Women Who Self‐Reported to Health Facilities for HIV Testing in Kenya: Analysis of 2022 Kenya Demographic and Health Survey

**DOI:** 10.1002/hsr2.70234

**Published:** 2024-12-04

**Authors:** Isaac Isiko, Kelly Taremwa, Simon Nyegenye, Aaron Mwesigwa, Reagan Muwanga Mutebi, Lenz Nwachinemere Okoro, Eneh Nchiek Edet, Catherine Chepkoskei Koech, Naya Gadzama Bulus, Jackson Micheal Asingwire

**Affiliations:** ^1^ Department of Community Medicine, Axel Pries Institute of Public Health and Biomedical Sciences NIMS University Jaipur India; ^2^ School of Public Health, College of Health Sciences Makerere University Kampala Uganda; ^3^ Department of planning and applied statistics, School of Statistics and Planning Makerere University Kampala Uganda; ^4^ Department of Pharmaceutical Sciences, Faculty of Health Sciences Marwadi University Rajkot India; ^5^ Department of Microbiology, Faculty of Health Sciences Marwadi University Rajkot Gujarat state India; ^6^ Department of Community Medicine David Umahi Federal University Teaching Hospital Uburu Nigeria; ^7^ Airport Clinic, Ministry of Health Nigeria; ^8^ Department of Sociology and Anthropology Maseno University Kisumu Kenya; ^9^ Department of Community Medicine Abubakar Tafawa Balewa University Bauchi Nigeria

**Keywords:** HIV in women, HIV stigma, HIV testing, HIV/AIDS disclosure, Kenya

## Abstract

**Background:**

This study aimed to determine the factors associated with feeling ashamed of disclosing HIV‐positive status among females who self‐reported to health facilities for HIV testing in Kenya.

**Method:**

This study used the Kenya Demographic Health Survey data set for 2022. A total of 18,506 women aged 15–49 years were selected from the sample clusters; 13,815 had ever tested for HIV and 332 had positive results for HIV. The chi‐squared test was applied to determine the association between the selected variables of interest and the outcome variable. Furthermore, to identify the explanatory variables that were associated with the outcome variable of interest, logistic binary regression was performed. *A p* > 0.05 and all statistical analyses were conducted using Microsoft Excel (xlsx) and STATA15.

**Results:**

The analysis included 332 women who had tested positive during the survey out of which 125(38%) women agreed to have felt ashamed to disclose their HIV+ status. Agreed to stigma (AOR = 1.92, 95% CI: 1.15, 3.22; *p* < 0.05) and being intimidated by health workers (AOR = 2.49, 95% CI: 1.05, 5.93; *p* < 0.05) were significantly associated with feeling ashamed of disclosing HIV+ status. The remaining variables, such as age category, residence, marital status, educational attainment, total number of children born, access to information, sex partners excluding spouses in the last 12 months, and number of lifetime sex partners, were not associated with feeling ashamed.

**Conclusion:**

Health stigmatization and intimidation Stigmatization had an almost two‐fold likelihood of causing shame in the disclosure of HIV status among females with HIV who were studied.

**Abbreviations**
2022 KDHS: Kenya Demographic and Health Survey data 2022
AIDS: acquired immune deficiency syndrome
AOR: adjusted odds ratios
ART: antiretroviral therapy
COR: crude odds ratios
DHS: Demographic and Health Survey
EPSEM: equal probability selection method
HIV: human immune virus
KNBS: Kenya National Bureau of Statistics
KPHC: Kenya Population and Housing Census
K‐HMSF: Kenya Household Master Sample Framework
MoH: Ministry of Health
PLWHA: people living with HIV/AIDS
PMTCT: prevention of mother‐to‐child transmission
USAID: United States Agency for International Development

## 1 Introduction

The global prevalence of HIV continues to decline, with Kenya achieving notable success in reducing the HIV prevalence among adults from 10% in the mid‐1990s to 4.5% in 2020 [1].

Kenya was one of the first African countries to report HIV infection, with the first case documented in 1984 [2, 3]. Strategies to reduce transmission have focused on increasing HIV testing and access to antiretroviral therapy (ART) [3]. In December 2014, the United Nations program on HIV/AIDS set the 90‐90‐90 strategy that aimed to ensure that 90% of HIV patients knew their status, 90% on treatment with antiretroviral therapy, and 90% have viral load suppression by 2020 [4]. However, barriers such as cost, access to care, risk perception, confidentiality concerns, and judgment from healthcare providers have hindered global testing rates [5]. In the US, one in seven people with HIV are unaware of their status [6], and hindrances are greater in southern states and rural areas due to stigma, lack of infrastructure, and poverty. Patients report high costs, uncertainty about accessing treatment, lack of risk awareness, and worries about character judgments as obstacles to testing. Racial and geographical differences have also been previously reported [6].

Youth and young adults have the lowest HIV testing rates, with only 55% of those aged 15–24 diagnosed in 2018 being aware of their positive status. Gender, race, sexual orientation, and socioeconomic status heavily influenced testing behaviors in this age group [7].

The female gender is reported to be at a higher risk than males It is estimated that half of the people living with HIV (PLWH) in sub‐Saharan Africa are women, especially of reproductive age (15–49 years) [8, 9]. This is attributed to the inequality of the female gender, poverty, discrimination, and stigma faced in family settings and healthcare facilities [10]. These factors have greatly influenced HIV testing and treatment behaviors of most child‐bearing women in sub‐Saharan countries, especially pregnant women [11]. Research involving teenage girls and young women in Rwanda and other sub‐Saharan African nations has revealed that pregnant women's attempts to test for HIV were thwarted by their inability to obtain access to health facilities, fear of stigma and discrimination, perceived lack of confidentiality from service providers, and failure to obtain permission from their husbands [12, 13].

The stigma associated with feeling ashamed remains the most significant drawback in HIV testing, prevention, and treatment. Anticipated stigma and fear of judgment from healthcare providers, families, and friends hinder HIV self‐reporting [14]. It correlates with adverse health effects for PLWH in the form of enacted (discrimination, prejudice), anticipated, internalized, and perceived stigma, thus negatively impacting testing and treatment decisions [15]. Stigma can also redefine a person from “whole and normal” to “tainted and devalued.” It is a fundamental contributor to delayed testing and poor medication adherence in PLWH. Discrimination towards HIV‐positive substance abuse patients further hinders access to care. Stigma and its consequences negatively impact pre and postdiagnosis decisions, psychological health, and quality of life. Previous studies recommend that the reduction of stigma and its related causes show a great increase in HIV healthcare‐seeking behaviors, including HIV testing and treatment [16, 17, 18]. PLWH suffer from isolation, depression, and suicidality risks following disclosure. Stigma can also lead to violence, rejection, a loss of quality of life, and broken relationships. Shame and judgment from family/friends also deter testing and disclosure [19].

Shame and guilt have also been reported by PLWH by family and friends as they get to know about their diagnosis, thereby hindering self‐reporting for HIV testing. In China and Vietnam, status disclosure risks unpredictable judgments. Surveys in Taiwan show fear of isolation, job/education loss, relationship dissolution, and excommunication following diagnosis [20, 21]. In Kenya, HIV is perceived to be an immoral disease. Research on HIV‐positive Kenyan children and caregivers has revealed that stigma, shame, and discrimination hinder care engagement through induced stress, depression, sadness, and loss of support. Over 20 per cent of PLWH across five European countries reported stigma‐related declines in daily functioning and cognitive impairment symptoms, such as anxiety, depression, and unemployment [22, 23, 24]. A study in Burundi characterized seven dimensions of HIV/AIDS‐related stigma suffered by people living with HIV/AIDS (PLWHA): physical violence, verbal abuse, marginalization, discrimination, self‐stigma, fear/insecurity, and stigma from healthcare workers, which together may induce shame in patients with HIV [25]. More than 8% faced physical violence, 67.5% verbal assaults, and 60.5% rejection/isolation. Discrimination included difficulty in finding jobs (3.5%), loan denials (5.3%), and rental evictions (6.1%). A total of 36.8% of participants reported self‐stigma‐like shame and suicide attempts. A total of 94.7% felt fear/insecurity regarding their diagnosis. These findings mirror those of other studies in Malawi, Burkina Faso, Uganda, and Kenya, showing the continued impact of stigma in the region. Stigma and shame not only affect PLWHA but also pose barriers to early testing, disclosure, and antiretroviral therapy (ART) initiation for new diagnoses [26].

While some studies show that disclosure can provide social/emotional support, adverse effects are more common overall. In addition to perceived stigma, the fear of testing positive also hinders self‐reported testing. This delays diagnosis and treatment raises the risks of opportunistic infections, costs, and HIV mortality, and may also worsen mental health [27, 28].

Despite the high general HIV awareness in Kenya, PLWHA, especially young females faces ongoing stigma with damaging social, personal, and treatment consequences. Qualitative research revealed that PLWHA faces lost opportunities, isolation, courtesy stigma toward caregivers/family, and internalized shame/self‐hatred [27, 29]. Therefore, there is an urgent need to address stigma to improve testing, diagnosis, treatment, and well‐being [29].

Therefore, this study assessed factors associated with feeling ashamed of disclosure of HIV‐positive status and other stigma consequences among females aged 15‐49 who self‐reported health facilities for HIV testing in Kenya using 2022 demographic/health data. Other factors analyzed included age, sex, residence, income, education, and social status. Relationships among factors that are determinants of HIV healthcare‐seeking behaviors are also described.

## 2 Methods and Materials

### 2.1 Study Setting and Design

Kenya is an East African country that borders Uganda, Tanzania, Sudan, Ethiopia, and Somalia. Kenya is administratively divided into eight provinces, namely, Nairobi, which is the capital city of Kenya, and the Coast, Eastern, Central, Northeastern, Western, Nyanza, and Rift Valley provinces, with a total of 70 districts and 47 counties that are divided into rural and urban strata. This resulted in 92 strata because Nairobi and Mombasa are completely urban provinces.

This research was conducted by analyzing secondary demographic and health survey data from Kenya in 2022.

### 2.2 Kenya Demographic and Health Survey Data 2022 (2022 KDHS)

The 2022 KDHS is a nationwide survey that provides the most current estimates of the nutrition, health indicators, and sociodemographics of the different households in Kenya [30].

The Kenya Demographic and Health Survey for 2022 was conducted and implemented by the Kenya National Bureau of Statistics (KNBS) in conjunction with the Ministry of Health (MoH) of Kenya. The 2021 KDHS is the 7th National Health Survey conducted in Kenya.

The data were collected from 17th February 17, 2022, to July 31, 2022. The ICF provided technical support during the survey through the Demographic and Health Survey (DHS) program funded by the United States Agency for International Development (USAID).

The 2022 KDHS sample was drawn from the master sample framework for Kenya households (K‐HMSF), which is currently used by the KNBS to conduct household‐based surveys in Kenya. The frame was based on the 2019 Kenya Population and Housing Census (KPHC) data, where 129,067 enumeration areas (EAs) were developed.

The 2022 KDHS survey included a sample design of two‐stage strata, where 1692 clusters were selected by the equal probability selection method (EPSEM) from the K‐HMSF [30]. The clusters were independently selected from each county, with 1026 clusters in rural areas and 666 clusters in urban areas. However, one cluster from Mandera County was not visited because of insecurity.

Four types of questionnaires were used as data collection tools: household, male, female, and biomarker questionnaires. The global DHS survey questionnaire was modified to meet the local needs of the survey.

The female, male, and household questionnaires were translated into Kiswahili for easier understanding by local respondents. All four questionnaires were programmed into a tablet computer to allow CAPI to ease the process of data collection.

The study used Kenya Demographic and Health Survey (KDHS) data for the period 2021 obtained with permission from the DHS website [30]. The KDHS uses a two‐stage cluster sampling design to select a nationally representative sample of households. In the first stage, clusters were selected from sampling frames using the most recently available census. In the second stage, households are selected from each cluster. There was a stratification of urban and rural areas for the different sampled clusters. This study used data extracted from women aged 15–49 years who indicated that they were HIV‐positive.

### 2.3 Variable Definitions

In our study, we included two types of variables: outcome and predictor/exposure. The outcome variable of interest was feeling ashamed of the HIV‐positive status disclosure, which was binary (being ashamed or not being ashamed). The exposure variables in the study included: whether the respondent is stigmatized(agreed to stigma, disagreed with stigma). The exposure variables in the study included: whether the respondent is stigmatized(agreed to stigma, disagreed with stigma). Agreed to stigma refers to women who acknowledged experiencing stigma due to their HIV‐positive status. This includes situations where someone else disclosed their HIV status without consent, people talked badly about them, and they felt verbally harassed, threatened, or insulted as a result of their HIV status.

On the other hand, “disagreed with stigma” refers to respondents who did not experience any form of stigma related to their HIV‐positive status. These individuals reported that no one disclosed their HIV status, no one talked badly about them, and they did not feel harassed or threatened due to their condition.

Other exposure variables included age category (adolescents, 15–24 years old and adults, 25–49 years old), residence (rural or urban), marital status (never married, married, living with a partner, and widowed/divorced/separated), education attainment (no education, primary and secondary and above), total children born(no children, 1–4 children, 5 or more children), access to information (no access to information, i.e., radio, reading newspapers, television, and access to information), number of sex partners excluding the spouse during the last 12 months (no sex partners and 1 or more sex partners) and whether the medical workers talked badly about the respondent (no intimidation, intimidated).

### 2.4 Data Analysis

**Figure 1:**
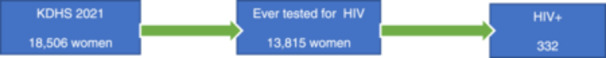
Selection of women aged 15–49 who tested HIV positive.

The statistical analysis focused only on women aged 15–49 years who reported that their test results indicated that they were HIV‐positive. The analysis considered the complex survey design, and weights were applied to ensure representativeness and to correct for nonresponse. A total of 332 women aged 15–49 who reported that they had been tested for HIV and whose results were positive were selected for analysis, as shown in Figure 1.

Crosstabulation of the outcomes with independent variables was performed using multiple frequency tables. At the bivariate level, the association between selected variables of interest and the outcome variable (feeling ashamed of HIV‐positive status) was estimated using a chi‐square test. Furthermore, logistic binary regression was performed to identify which explanatory variables were associated with the outcome variable of interest (feeling ashamed because of HIV‐positive status). Only factors with *p*‐values less than or equal to 0.05 were included in the multivariable model. The results were accepted at 95% confidence intervals. All statistical analyses were conducted using Microsoft Excel (xlsx) and STATA15.

**Figure 2:**
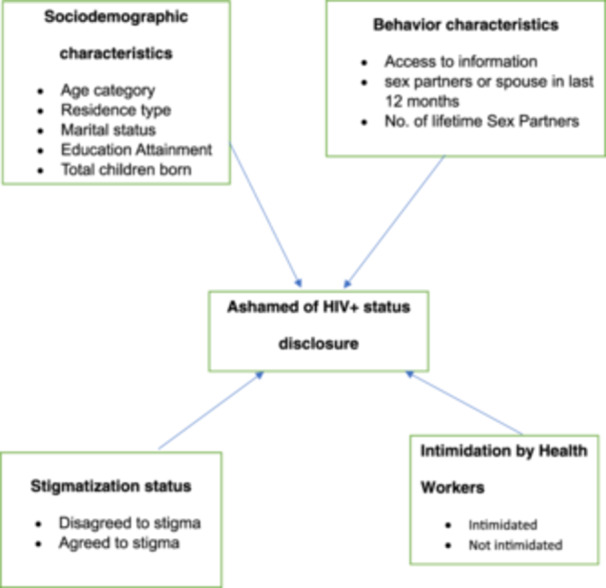
Conceptual framework [31].

The conceptual framework in Figure 2 shows the different background characteristics of HIV+ women in Kenya who felt ashamed of disclosing their HIV+ status [31].

## 3 Results of Analysis

After cross‐tabulation between women feeling ashamed and women with selected background characteristics in Table 1, the overall prevalence of feeling ashamed among HIV‐positive women was 38% (125). Significant differences were observed based on the selected background characteristics. Stigmatization status was the only covariate significantly (*p* = 0.015) associated with women being ashamed of HIV‐positive status (*p* < 0.05). It was also observed that women experiencing stigma were significantly more likely (52.9%) to be ashamed of their HIV‐positive status than women with no stigma (47.1%). The analysis further revealed that women who did not feel intimidated by health workers reported higher levels of shame (87%) than those who were not intimidated (13%). The results also revealed that women with 1–4 children (62.7%) and those with five or more children (30%) tended to report higher levels of shame than women without children (7.3%). Furthermore, women who lived in rural areas (68.9%) experienced greater shame related to their HIV status than those who lived in urban areas (31.1%). It was also observed that adolescent HIV women (9.1%) had less chance of feeling ashamed than adults (90.9%).

**Table 1:** Women who felt ashamed of disclosure of HIV+ status by selected background characteristics.

Variables	Ashamed of HIV+ status disclosure? (*N* = 332)				*p* value
	Disagreed		Agreed		
	*n* = 207	Percentage (100%)	*n* = 125	Percentage (100%)	
**Stigmatized?**					**0.015**
with no stigma	131	63.5	59	47.1	
with stigma	75	36.5	66	52.9	
**Age category**					0.85
Adolescents	17	8.3	11	9.1	
Adults	190	91.7	114	90.9	
**Residence**					0.735
Urban	69	33.6	39	31.1	
Rural	137	66.4	86	68.9	
**Marital status**					0.616
Never married	27	13	12	9.8	
Married/living with partner	105	51	72	57.1	
Widowed/Divorced/Separated	74	36	42	33.1	
**Education Attainment**					0.838
No education	13	6.1	6	4.5	
primary	132	64	83	66.5	
Secondary+	62	29.9	36	29.1	
**Total children born**					0.705
0	12	5.7	9	7.3	
1–4	141	68.2	79	62.7	
5 and above	54	26.2	38	30	
**Access to information**					0.982
No Access	20	9.7	12	9.8	
Has Access to information	187	90.3	113	90.2	
**sex partners ex. spouse−12 months**					0.876
None	154	74.4	92	73.5	
One or more	53	25.6	33	26.5	
**Intimidated by health workers**					0.523
Not intimidated	186	90.2	109	87	
Intimidated	20	9.8	16	13	

### 3.1 Logistic Regression

After adjustment, agreed‐upon stigma and intimidation by health workers were significantly associated with feeling ashamed *(p* < 0.05). The results presented in Table 2 show that agreement with stigma (AOR = 1.92, 95% CI: 1.15, 3.22, *p* < 0.05) and being intimidated by health workers (AOR = 2.49, 95% CI: 1.05, 5.93, *p* < 0.05) were the only statistically positively associated factors associated with agreeing to shame by the end of the follow‐up. The likelihood of feeling ashamed was 1.9 times greater (AOR = 1.92; 95% CI = 1.15–3.22) for HIV‐positive women who experienced stigma than for those who did not experience stigma. An HIV‐positive woman who reported being intimidated by a health worker had 2.5 greater odds of feeling ashamed (AOR = 2.49; 95% CI = 1.05−5.93) than women who did not feel intimidated. However, age category, residence, marital status, educational attainment, total number of children born, access to information and sex partner status excluding one's spouse in the last 12 months, were not associated with feeling ashamed.

**Table 2:** Logistic regression analysis of factors associated with feeling ashamed of disclosure of HIV+ status among self‐reported women in Kenya.

Variables	Categories	Bivariate	Multivariate
		UOR (95% CI)	AOR (95% CI)
**Stigmatization status**	Disagreed to stigma	1	1
	Agreed to stigma	**2.00** * **(1.28, 3.14**)	**1.92** ** **(1.15,3.22)**
**Age category**	Adolescents	1	1
	Adults	0.93 (0.43, 2.01)	1.08 (0.36,3.24)
**Residence type**	Urban	1	1
	Rural	0.98 (0.62, 1.56)	0.97(0.59,1.62)
**Marital status**	Never married	1	1
	Married/living with partner	1.08 (0.53, 2.20)	2.02 (0.72,5.63)
	Widowed/Divorced/Separated	1.04 (0.49, 2.19)	1.65 (0.63,4.33)
**Education attainment**	No education	1	1
	primary	0.93 (0.36, 2.37)	1.33 (0.47,3.77)
	Secondary+	1.14 (0.43, 3.04)	1.62 (0.53,4.92)
**Total children born**	0	1	1
	1–4	0.94 (0.39, 2.28)	0.99 (0.29,3.42)
	5 and above	1.24 (0.49, 3.13)	1.48 (0.39,5.52)
**Access to information**	No Access	1	1
	Has Access to information	1.06 (0.55, 2.02)	1.19 (0.58,2.46)
**Sex partners/spouse last 12 months**	None	1	1
	one or more	1.01 (0.61, 1.68)	1.19 (0.58,2.46)
**Intimidated by Health workers**	Not intimidated	1	1
	Intimidated	**3.07** ** **(1.42, 6.64**)	**2.49** ** **(1.05,5.93)**

* *p* < 0.01.

** *p* < 0.05.

### 3.2 Stratified Analysis

Based on the results in Table 3, neither the Breslow‒Day (BD) nor the Mantel–Hanszel statistics (tests of homogeneity) were significant (*p* = 0.1171, *p* = 0.1171); hence, intimidation was not an effect modifier in the relationship between stigma and agreeing with shame. The Mantel–Haenszel combined odds ratio for the relationship between stigma and agreeing to shame among study participants (HIV‐positive women) was 1.69 (95% CI: 1.05–2.73). The results indicated a moderate connection between experiencing stigma and feeling ashamed in both groups: those who felt intimidated and those who did not. Specifically, the chances of feeling ashamed due to stigma were found to be 1.7 times greater (with a range of 1.05 to 2.73) in both groups—those who felt intimidated and those who did not.

**Table 3:** Association between stigma, intimidation status, and agreement to shame among HIV‐positive women.

Stratum	Crude		M‐H Combined		*p* values
	OR	95% CI	OR	95% CI	
Not Intimidated	2.00	1.24−3.22	1.69	1.05−2.73	0.1171 (M‐H) 0.1171 (B‐D)
Intimidated					

*Note:* M‐H is the Mantel–Hanszel test, B‐D is the Breslow‒Day test, and OR is the odds ratio

## 4 Discussion

This study assessed and identified the factors associated with feeling ashamed of disclosure of HIV‐positive status among females of reproductive age who self‐reported to health facilities for HIV testing in Kenya. This study provides germane insights into the multipronged and intertwined nature of HIV disclosure experiences among the study population, with slightly more than one‐third of the respondents feeling ashamed of disclosing their HIV‐positive status. Although these findings are greater than those reported in rural South Africa [1], Kenya [2], Mozambique [3], and East Africa [4], they provide an estimate of the public health burden of poor disclosure and its resultant effect among PLHIV and will lead to long‐term policy shifts and advocacy efforts toward effective behavior change communication in the control of HIV. Possible reasons contributing to this disparity include cultural differences, variations in healthcare support, access to education, and differences in study methodologies across different study settings and populations. Understanding these factors is crucial for interpreting and addressing emotional experiences related to HIV disclosure among women in diverse settings.

The study also identified intimidation by health workers as a predictor of feeling ashamed of the disclosure of positive HIV status among the study respondents. This finding differs from that of a study performed in South Africa that identified discrimination from family as a major predictor of poor disclosure of HIV status [32]. This may be explained by the relevance of strong family ties in ensuring unalloyed family support systems in providing care and other essential services. This may not be far‐fetched, as feeling ashamed about disclosing one's HIV status may pose a deterrent to avoiding possible discrimination from the family, which may compromise family ties with its attendant fragile family support systems that may hamper access to care and support from family members. On another note, the findings of the study are consistent with those of a study on combating HIV stigma in healthcare settings [33, 34, 35]. This underscores the critical role of healthcare settings in shaping the experiences of individuals living with HIV. Interventions targeting healthcare providers should prioritize creating supportive and nonintimidating environments to encourage open disclosure.

As noted in this study, stigmatization and intimidation by health workers can deter individuals from pursuing HIV testing, resulting in delayed diagnosis and a reluctance to disclose their status [36]. This reluctance hampers early intervention and treatment initiation, leading to the progression of HIV infection and an elevated risk of transmission within communities. Furthermore, this approach creates missed opportunities for essential prevention of mother‐to‐child transmission (PMTCT) interventions, heightening the risk of vertical transmission [37, 38, 39, 40]. Additionally, the profound mental health implications of stigma‐induced shame manifest as heightened stress, anxiety, and depression among HIV‐infected individuals living with HIV. This, in turn, negatively impacts their overall well‐being and compromises their adherence to crucial treatment regimens.

The study further showed that none of the variables were confounding factors for the association between stigma and feeling ashamed [39]. The absence of confounding effects from demographic factors such as age category, residence, marital status, educational attainment, total number of children born, access to information, sex partners (excluding spouses) in the last 12 months, and the number of lifetime sex partners is noteworthy. This emphasizes the robustness of the identified factors (stigmatization and intimidation by health workers) and their independent impact on the outcome (feeling ashamed), which is an edge this study has over many studies on the disclosure of HIV status [5, 6, 7, 32].

As the multifaceted issue of HIV‐related shame is being addressed, there is a need for communities and healthcare settings to be reached through campaigns focused on destigmatization. Additionally, emphasis needs to be placed on healthcare providers' education and training to ensure patient‐centered care devoid of intimidation. This study also contributes valuable insights into ongoing efforts to enhance the psychosocial well‐being of individuals living with HIV. By addressing and mitigating stigma and intimidation, a more compassionate and supportive environment can be created for those affected can be created [38].

## 5 Conclusion

Stigmatization and intimidation by health workers were identified as factors associated with feeling ashamed of disclosure of HIV‐positive status in this study, as stigmatization had an almost two‐fold likelihood of causing shame in disclosure of HIV status among females with HIV. The association between stigmatization and feelings of shame about disclosing HIV status aligns with global trends, as reported in studies from various regions (such as Uganda [5], southwestern Texas [6], and Ghana [7]), which have consistently identified stigma as a major barrier to opening up HIV disclosure.

## 6 Limitations of the Study

Since HIV/AIDS is a very sensitive topic of study, there is a high chance that the respondents modified their responses for fear of shame and stigmatization after exposure to HIV. The study was also carried out only on female data within the KDHS, which strongly limits the number of observable features, thus slightly affecting the results of our study. The study findings cannot be used to conclude the whole population of Kenya, as some clusters, such as Mandera County, were left out due to inaccessibility due to insecurity in these areas, and the study was based purely on data from women; hence, the findings cannot be used to distinguish other groups of people, such as men and children, within Kenyan communities

After testing for confounding, none of the variables met the defined threshold of a 10% change, indicating that no confounding factors were present.

## Author Contributions

I.I. conceived the idea and drafted the manuscript. S.N., C.T., and I.I. performed the analyses, interpreted the results, and drafted the subsequent manuscript versions. A.M., R.M.M., L.N.O., E.N.E., B.N.G., J.M.A., and I.I. wrote the introduction and discussion sections of the manuscript. C.C.K., A.M., and I.I. reviewed the first draft and all the corresponding versions of the manuscript. All authors have read and approved the final version of the manuscript.

## Ethics Statement

The data set was accessed through the DHS Program website https://www.dhsprogram.com/data/available-datasets.cfm. by drafting the topic and abstract of the research for which the data were to be used.

The Demographic and Health Survey also ensured that personal details such as names and addresses were not identified in all the data before permitting us to access the data. The ICF IRB accepted our application and granted us approval to download the 2022 Kenya Demographic and Health Survey (2022 KDHS).

For all participants from whom the DHS enumerators collected the data, verbal informed consent was obtained, and if the participants were minors, their parents provided consent on their behalf. Details can be found in the 2022 KDHS.

## Conflicts of interest

The authors declare no conflicts of interest.

## Data Availability

The data used in our study can be accessed by logging onto the DHS website upon request. (https://www.dhsprogram.com/data/available-datasets.cfm).
